# A survey of school’s preparedness for managing anaphylaxis in pupils with food allergy

**DOI:** 10.1007/s00431-020-03645-0

**Published:** 2020-04-05

**Authors:** George Raptis, Mercedes Perez-Botella, Rebecca Totterdell, Konstantinos Gerasimidis, Louise J. Michaelis

**Affiliations:** 1 Department of Infectious Diseases, Immunology & Allergy, Royal Hospital for Children, Queen Elizabeth Hospital, Glasgow, UK; 2 University of Central Lancashire, Preston, UK; 3 School of Medicine, Dentistry & Nursing, Human Nutrition, University of Glasgow, Glasgow, UK; 4 Department of Immunology, Infectious Diseases & Allergy, Great North Children’s Hospital, Newcastle, UK

**Keywords:** Schools, Anaphylaxis, Surveys and questionnaires, Organisational policy

## Abstract

Allergic diseases are on the increase and can affect the child’s well-being. The aim of this survey was to assess regional schools’ preparedness in dealing with anaphylaxis following the publication of national and international guidelines for schools in 2014. The survey was developed in 2015 and distributed to schools in Cumbria, North West England, UK between 2015 and 2016. Only 47% of the respondents (95% CI, 39–57%) felt confident to manage anaphylaxis. Schools without allergic pupils were significantly less likely to have a standard management protocol in place for emergencies compared to those with allergic pupils (*p* < 0.001). The majority of the schools indicated that further training was needed (81% (95% CI, 74–88%).

*Conclusion*: At the time of the survey, schools’ preparedness in the region, did not meet safety standards recommended by national and international organisations. Although schools have shown eagerness in accessing training in the management of anaphylaxis, tailored training for schools is not yet widely available. There is now an urgent need to design feasible training strategies that create a safe environment for allergic pupils across all UK schools.

**Table:** 

**What is Known:** *• One quarter of the severe allergic reactions take place for the first time while at school with some of them being fatal.* *• School staff is ill-prepared in the management of anaphylaxis. Access to formal training is not widely available.*	
**What is New:** *• School staff remains unconfident in managing the severe allergic child.* *Training in the management of anaphylaxis is scarce, and when available, it does not offer the required depth to cover the holistic needs of allergic pupils.* *• Schools would welcome generic adrenaline autoinjectors and a national policy with central funding which would describe step by step the necessary measures for the management of anaphylaxis.*

**What is Known:**

*• One quarter of the severe allergic reactions take place for the first time while at school with some of them being fatal.*

*• School staff is ill-prepared in the management of anaphylaxis. Access to formal training is not widely available.*

**What is New:**

*• School staff remains unconfident in managing the severe allergic child.* *Training in the management of anaphylaxis is scarce, and when available, it does not offer the required depth to cover the holistic needs of allergic pupils.*

*• Schools would welcome generic adrenaline autoinjectors and a national policy with central funding which would describe step by step the necessary measures for the management of anaphylaxis.*

## Introduction

In the UK, allergic diseases have reached epidemic levels and some of these diseases are becoming more complex, life threatening and at times fatal [18]. There are misunderstandings and controversies around the diagnosis and management of anaphylaxis [2]. What is clear is that children are at higher risk of severe reactions due to increased exposure to allergens in schools, with 25% of the food allergic reactions occurring during school activities [2, 17, 18]. Fatalities at school from anaphylaxis in the UK have led to concerns over the level of school staff preparedness in managing the severe allergic child [27].

Available data suggests that UK schools are not prepared to deal with severe allergic reactions; less than half have a trained teacher and adrenaline autoinjectors (AAIs) are only available in 12% of the schools [23]. Other European countries display a similar school preparedness pattern. A large survey of Italian schools showed that their staff had limited knowledge in the risks associated with food allergy, under appreciation for the psychosocial impact on pupils and that they believed administering adrenaline could pose a risk to pupils. They also reported a lack of specialist training to be the main difficulty in managing anaphylaxis at school [21]. Likewise, a survey conducted in France reported a low number of personalised allergy action plans (PAAP) being in place in schools when compared with the prevalence of food allergies in school-aged children. They also reported the lack of guidance with regard to the essential contents of emergency kits and where they should be stored [22]. Since almost one quarter of children with allergies have their first allergic reaction at school [16], it is imperative that staff is trained to prevent, recognise and treat severe allergic reactions irrespective of whether they currently have pupils with such medical history.

Law in the UK now permits school staff to administer an emergency AAI to any child who has been assessed as being at risk of anaphylaxis [11]. The current proposal of provision of spare AAIs in nurseries and schools may address availability issues when the child’s own device is inaccessible. However, the responsibility is placed on the school to implement their own allergy policy, which introduces clinical governance issues and a risk of disparity between schools [11, 15, 27].

A nationwide policy on the prevention and management of severe allergic reactions at schools could potentially alleviate some of the current shortcomings of UK schools’ preparedness. In 2005, Ontario’s government approved the ‘Act to Protect Anaphylactic Pupils’ (Sabrina’s Law), the first legislation of this type in the world, following a fatal anaphylaxis reaction at school. It requires head teachers to work in partnership with health professionals to develop and maintain an anaphylaxis policy in every school [26]. Having this legislation in place has been shown to improve efforts made by schools to support pupils with food allergies, although further resources may be required to prepare school staff to manage anaphylaxis [7].

The publication of the European Academy of Allergy and Clinical Immunology (EAACI) ‘Food Allergy and Anaphylaxis Guidelines’ [17] in 2014 and the UK statutory guidance ‘Supporting pupils at school with medical conditions’, updated in 2017 [9], promote the development of (a) school policies (led by the head teacher) for the management of the allergic child in school in collaboration with a multiprofessional specialist team and (b) a strong communication network with all the stakeholders [9, 17].

To implement a successful policy and reduce delayed response to anaphylaxis, schools require appropriate training and support in allergy management by accredited professionals.

Current guidelines state that all school staff should receive training in how to prevent, recognise and respond to anaphylaxis [9, 17, 18]. However, what is available at schools seems to be inconsistent and irregular. Previous studies evaluating the effect of training interventions have been successful at improving participant knowledge, self-rated confidence and reducing the clinical risk of reactions, which remain significant after 4–12-week follow-ups [12]. Yet, access to such training for schools is poor as standardised initiatives across institutions with secured funding for implementation seem to be lacking. There is a clear need to determine the level of school preparedness to manage a child with severe allergies and revise current training practices. A recent publication calls for urgent action in schools with mandatory measures. They propose more specific guidance for schools, secured funding for both anaphylaxis training for school staff and pupils and spare AAIs [27].

The aim of this survey was to assess school preparedness in dealing with allergic reactions in primary schools in Cumbria, North West England, following the publication of national and international guidelines on the management of anaphylaxis at schools in 2014. The survey was developed in 2015 and distributed to schools between 2015 and 2016 [9, 17].

## Materials and methods

### Questionnaire design

There is currently no validated questionnaire (or any other tool) to assess school preparedness in the management of allergic reactions. A 38-item, structured questionnaire was developed based on national and international recommendations [1, 4, 6, 9, 10, 13, 17, 19, 23]. This was piloted amongst a multidisciplinary team of nine professionals, including three community nurses, two allergist specialists, two paediatricians and two administration staff, to ascertain clarity and relevance of the questions. Two out of the county teachers were also asked to complete and give feedback which was incorporated in the final version of the questionnaire. The questionnaire was further piloted amongst head teachers in order to assess its suitability and its content validity. The questionnaire was revised based on their comments.

The questionnaire included the following sections:

a. Demographic data: this section was designed to capture the school’s characteristics (type of school and funding stream were collected from the published List of Schools and other Educational Establishments) [8].

b. ‘Understanding the school’: schools were asked whether they have allergic pupils, type and severity of allergies.

c. Allergic management training arrangements in the school; training offered by and to schools: questions in this section aimed to capture data on current training arrangements for staff as well as the training they deliver to pupils and their families on the management of allergies.

d. Preventative measures: this section investigated current policies in place to prevent food allergic reactions mainly. Schools were asked to skip this set of questions if they did not have registered pupils with allergies.

e. Further support for the school when caring for children with allergies: schools were given the opportunity to express their needs on further training and their views on the need for national guidelines for the management of severe allergies at school.

Most of the questions were designed as dichotomous or Likert scales and free text options were available for some questions. At the end of the survey, participants were invited to give comments on any issues they felt needed addressing related to the care of the allergic child at school. This was an open-ended question. The Cardiff’s Teleform information capture system [5] was used to design the questionnaire, collect and transfer the data.

### Inclusion and exclusion criteria

All schools in Cumbria (*n *= 315) were invited to participate in this study; however, only the data from the primary schools (87%, *n* = 275) are presented here. Data from secondary school respondents was removed from the analysis due to the small sample size (*n* = 22). Special needs schools, academies, colleges and nurseries were excluded. The most recent summary list of schools and other educational establishments produced by the local education authorities (LEA) of Cumbria at the time of the study was used to identify and invite schools to participate in the survey.

### Posting of the questionnaires

A package containing a cover letter explaining the aim of the study, the questionnaire and a stamped, pre-addressed envelope was sent to all eligible schools. The letter was addressed to the head teachers, but it was indicated within the letter that it may be forwarded to those who were better placed to answer the questionnaire, if needed. They were invited to complete the questionnaire within 4 weeks. Non-responders were sent two reminders on week 5 and 6. The survey was completed on March 2016.

### Statistical analysis

Categorical data were presented with counts and frequencies and associated 95% CI. Some of the survey items had missing data; therefore, the number of respondents for each item was calculated and the valid percentage reported. ‘Do not Know’ responses were excluded from the analysis.

Chi-squared test was used to compare categorical data between two groups (e.g. schools with pupils at risk of anaphylaxis and those without). A probability level of < 0.05 (*p* < 0.05) was considered statistically significant. Analysis was performed with IBM SPSS Statistics v25.

### Ethical approval

Permission to send out the questionnaire was sought from the LEA which advised that the decision to participate should be made by each individual school. Schools were given the study information pack to read and asked to participate voluntarily. Completion and return of the questionnaire was taken as an informed consent.

## Results

A total of 157 (57%) primary schools responded to this survey. No differences in school characteristics were found between the respondents and non-respondents. They were from diverse socio-economic catchment areas; the mean English Index of Multiple Deprivation decile score was 5.46 and 5.38 for respondents and non-respondents respectively. The ‘do not know’ answers were removed from the analysis as they represented a very small percentage of the overall responses (between 3 and 10%).

### Understanding the school: prevalence of severe allergies

A total of 24,174 pupils attended the 157 schools who responded to the survey. Of these, 165 pupils were known to have a history of anaphylaxis or they were at risk of severe allergic reactions and they had an AAI prescribed, a prevalence of 0.7% (95% CI, 0.59–0.81%). The number of reported allergic pupils per school ranged from 1 to 12, (median = 1, IQR = 1–2). Eighty-nine schools (57% (95% CI, 49–65%)) reported they had pupils who had experienced severe allergic reactions in the past. From 86 schools who responded to the sub-question on whether a PAAP was in place for such pupils, 77 schools (90%, (95% CI, 81–95%)) confirmed they had. Furthermore, 71 (46%) schools had pupils at risk of anaphylaxis and carried an AAI.

### Existing guidelines and protocols in school for the management of severe allergic reactions

**Fig. 1:**
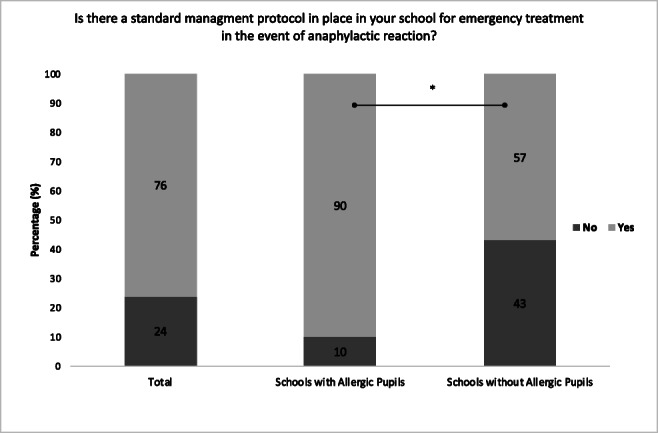
The existence of a standard management protocol in the school for the emergency treatment of a severe allergic reaction. This figure presents the responses from all schools and from the subgroups ‘with’ and ‘without registered severely allergic pupils’. Statistically significant differences are indicated with asterisks (* *p* < 0.001, compared with chi-squared test)

One hundred fifty-two schools responded to this question. One hundred eleven schools (76% (95% CI, 68–83%)) had a standard management protocol in place for emergency treatment in the event of a severe allergic reaction (Fig. 1). Of the schools with registered severe allergic pupils, 77 (90% (95% CI, 81–95%)) had such a protocol. Twenty-six out of the 60 schools with no registered pupils at risk of anaphylaxis (43% (95% CI, 31–57%)) did not have a management protocol. The difference between schools with and without pupils at risk of anaphylaxis reached statistical significance (*p* < 0.001, Fig. 1).

### School preparedness for severe allergic reactions

The responses from schools regarding their preparedness are displayed in Table 1. Respondents did not seem to be as prepared to manage severe allergic reactions in pupils without a prior history of allergies (61% (95% CI, 50–71%)). There was no difference in response between schools that had allergic pupils and those without.

**Table 1:** School preparedness for severe allergic reactions. This table depicts the preparedness measures in place at each school. Results for schools with and without allergic pupils are compared with the chi^2^ test. No significant differences were found

Respondents (*n* (%)/95% confidence interval (%))								
Question Has your school prepared for allergy emergencies by?	Total sample			Schools with allergic pupils		Schools without allergic pupils		*X*^2^ test*
	Yes	No	*n*	Yes	No	Yes	No	
Setting up communication systems within the school that are easy to use in emergencies?	96 (94) CI 88–98	6 (6) CI 2–12	102	73 (96)	3 (4)	23 (89)	3 (11)	2.02 *p* = 0.16
Identifying the role of each staff member in an allergy emergency?	78 (82) CI 73–89	17 (18) CI 11–27	95	59 (82)	13 (18)	19 (83)	4 (17)	0.01 *p* = 0.94
Preparing for allergic reactions in children without a prior history of allergies?	54 (61) CI 50–71	35 (39) CI 29–50	89	42 (61)	27 (39)	12 (60)	8 (40)	0.01 *p* = 0.94
Documenting the response of the staff to an allergy emergency?	72 (82) CI 72–89	16 (18) CI 11–28	88	55 (82)	12 (18)	17 (81)	4 (19)	0.01 *p* = 0.91

*X^2^ comparison between schools with and without allergic pupils, values significant if *p* < 0.05

### Generic provision of adrenaline autoinjector and management plan

From all the questionnaires returned, 100 schools answered the question regarding the generic provision of AAIs (64% response rate). Ninety-four percent (95% CI, 88–98%) stated they either ‘agree’ or ‘strongly agree’ with the generic provision of AAI and an anaphylaxis management plan to be kept in the school for any pupil who might develop a severe allergic reaction. Although not statistically significant (*p* = 0.09), schools without allergic pupils were more likely to strongly agree compared with those with allergic pupils registered (55.6% vs 30.2%).

### School training needs

Out of 112 respondents, 53 (47% (95% CI, 39–57%)) reported feeling confident to manage anaphylaxis. Although there was no significant difference in the level of confidence (*p* = 0.10), schools with allergic pupils rated themselves more confident compared with those without registered allergic pupils (52.6% vs 36.1%) respectively.

A significant number of schools (114 out of 140 respondents) felt that further training was needed (81% (95% CI, 74–88%)), while the majority stated that face-to-face training was preferable (70 out of 116 respondents for this particular sub-question; 60% (95% CI, 51–69%)). Responses for each question were similar between schools with and without pupils at risk of anaphylaxis (*p* = 0.53 and *p* = 0.23 respectively).

### Preventative measures

Table 2 lists the responses to the different questions on preventative measures in place at schools. There were some missing responses on the ‘preventative measures’ section. The total number of the recorded responses is reported in Table 2. From 104 respondents, only half of these schools (49% (95% CI, 39–59%)) offered special supervision for high-risk children at meal times.

**Table 2:** Preventative measures with sub-analysis. This table shows the policies in place to prevent allergic reactions in schools. Results for schools with and without allergic pupils are compared with the chi^2^ test. A significant difference was found only for the ‘no eating policy on transport to and from school’

Respondents (*n* (%)/95% confidence interval (%))								
				Schools with allergic pupils		Schools without allergic pupils		*X*^2^ test*
Question	Yes	No	*n*	Yes	No	Yes	No	
Is there guidance for staff handling food on the prevention of anaphylaxis?	82 (79) CI 70–86	22 (21) CI 14–30	104	60 (82) CI 73–91	13 (18) CI 9–27	22 (71) CI 55–87	9 (29) CI 13–45	1.64 *p* = 0.20
Is there special supervision for high risk children at meal times?	51 (49) CI 39–59	53 (51) CI 41–61	104	40 (51) CI 40–62	39 (49) CI 38–60	11 (44) CI 25–63	14 (56) CI 37–75	0.33 *p* = 0.56
Is there a no food-sharing policy for children at your school?	72 (63) CI 54–72	42 (37) CI 28–46	114	53 (65) CI 55–75	29 (35) CI 25–45	19 (59) CI 42–76	13 (41) CI 24–58	0.27 *p* = 0.60
Is there a no eating utensil policy for children at your school?	49 (45) CI 35–55	60 (55) CI 45–65	109	37 (47) CI 36–58	41 (53) CI 42–64	12 (39) CI 22–56	19 (61) CI 44–78	0.68 *p* = 0.41
Is there a no nut policy for children at your school?	61 (55) CI 45–64	51 (45) CI 36–55	112	48 (59) CI 48–70	33 (41) CI 30–52	13 (42) CI 25–59	18 (58) CI 41–75	2.71 *p* = 0.10
Are you aware of the Food Standards Agency new food information regulation regarding non-packaged foods?	69 (66) CI 56–75	35 (34) CI 25–44	104	46 (62) CI 51–73	28 (38) CI 27–49	23 (77) CI 62–92	7 (23) CI 8–38	2.01 *p* = 0.16
Have relevant teaching sessions (i.e. cooking classes) been reviewed to ensure no potential trigger foods for anaphylaxis are used?	70 (68) CI 58–77	33 (32) CI 23–42	103	50 (67) CI 56–78	25 (33) CI 22–44	20 (71) CI 54–88	8 (29) CI 12–46	0.21 *p* = 0.65
Is there a ‘no eating on policy on transport to and from schools?	37 (48) CI 37–60	40 (52) CI 40–64	77	21 (39.6) CI 27–53	32 (60.4) CI 47–72	16 (66.7) CI 47–82	8 (33.1) CI 18–53	4.84 *p* = 0.03

*X^2^ comparison between schools with and without allergic pupils, values significant if *p* < 0.05

Several schools without registered allergic pupils also answered the questions on preventative measures. This could be because they misread the survey guidance or because they wanted to share their practice in this area. Comparative sub-analysis (Table 2) showed that similar practices on anaphylaxis preventative measures were in place between schools with and without registered pupils with allergies.

### Need for further information and guidance on the management of severe allergic reactions

Out of 147 respondents, 128 schools (87% (95% CI, 81–92%)) reported that they would like further information on the management of severe allergic reactions. Similarly, 124 out of 138 respondents (90% (95% CI, 84–94%)) believed that a national school policy is needed to manage anaphylaxis appropriately and safely at school. There was no significant difference between schools with allergic pupils and those without (*p* = 0.3). It was frequently reported in the last, open-ended question that regular in-house training for all school staff was needed.

## Discussion

The aim of this survey was to assess school preparedness in dealing with allergic reactions in primary schools. The survey showed that 57% of the schools reported that they had pupils with previous severe allergic reactions, while just under one in two (46%) schools confirmed they had pupils at risk of anaphylaxis and carry an AAI. However, around 25% of severe allergic reactions at school occur in pupils with no history of allergies, so it is suspected that the actual prevalence might be higher [15, 16, 18].

This survey revealed that less than half of the schools felt confident in managing severe allergic reactions. Similar results have been reported previously and it is agreed that staff-perceived confidence is a good indicator of the school preparedness to deal with allergy emergencies [20, 23, 24, 29]. This lack of confidence stands to reason, since school training is limited with regard to regularity and quality; the fact that training is offered upon request, and is not mandatory, places the responsibility on the school to acquire training, thus reducing the motivation for schools without registered allergic pupils.

Regional study days covering several medical conditions (such as epilepsy, diabetes and allergy) are offered to all schools (with one teacher attending who then cascades the information). However, it is plausible to assume that the teachers may feel overwhelmed with an overload of information on relatively unfamiliar topics to them. A brief talk on allergy may also not be sufficient in generating confidence on the subject. Indeed, 4 years later, to the best of our knowledge, the school training format has not changed in the survey’s catchment area or the areas currently being studied (across North West of England and Scotland) [27]. Patient organisations such as Anaphylaxis Campaign are now leading this area and a number of schools across the country are receiving guidance and training from them [3]. It is anticipated that following this initiative funded by the Anaphylaxis Campaign, an improvement on school staff preparedness and confidence will be achieved. However, these training programmes should be government led so they become widely available and mandatory for all schools [27]. The vast majority of schools felt that further training was needed, and face-to-face training seemed to be preferable. Schools without allergic pupils were just as likely to report this as schools with registered allergic pupils, suggesting a need for regular training sessions across the board. With the recent reduction of school nurses from most UK schools, it is likely that this will have further impact on school preparedness [14, 25]. Schools regularly reach out for help from health professionals; however, resources are not always available to offer support and guidance. Future work should focus on how this training should be delivered to schools in a feasible and effective manner.

Based on the findings of the present survey, schools with or without allergic pupils were likely to have similar preventative measures, albeit below accepted standards in both cases. For instance, allergic pupils should be supervised during meal times, food and drinks that are offered by the school should be clearly labelled, emergency medication should be stored in easily accessible, central location and regular, comprehensive practical training sessions in the emergency response to anaphylaxis should be available to all school staff. Since preventative measures are the cornerstone in anaphylaxis management at school, considerable work is still needed to reach the necessary standards. There are now available national and international guidelines; however, such recommendations are yet to be implemented [11, 17, 27]. Further guidance to schools should be offered and steps towards this have been made both from clinical services and AAI manufacturers, but the uptake from the schools is not yet satisfactory [27].

Schools agree that a national policy for the management of these pupils is required. They would also like generic AAI and management plans to be kept at school for any pupil who might develop a severe allergic reaction for the first time while at school. Schools without allergic pupils were just as likely to ‘strongly agree’ or ‘agree’ with this statement as schools with allergic pupils. The recent guidance from the Department of Health in the UK allows the generic use of spare AAIs only for those who have been diagnosed as at risk of anaphylaxis, have medical authorisation and consent from parents [11]. At present, governance and safety issues along with the cost implications of purchasing spare pens by schools have prevented them to have in place emergency medication for severely allergic pupils.

It is encouraging that schools have a PAAP for pupils at risk of anaphylaxis and a standard management protocol in case of a severe allergic reaction. It has been shown that a PAAP and management protocols can reduce the frequency and severity of allergic reactions [13, 28]. However, the lack of a standard management protocol for almost half of the schools which currently do not have pupils at risk of anaphylaxis is concerning. Furthermore, 40% of schools who did not have any registered pupils with severe allergies confirmed they were not prepared to deal with an unexpected severe allergic reaction. This is concerning as children are at risk of having their first severe reaction at school with 25% of reactions occurring in pupils with no previous history [16]. This reinforces the need to develop effective strategies and initiatives that will encourage schools without allergic pupils to improve their awareness and preparedness towards anaphylaxis.

Overall, the preparedness issues that were found in primary schools were also seen in secondary schools; detailed comparisons on specific areas such as preventative measures were not warranted due to the small sample size and the difference in feasible approaches employed between the two establishments, such as no food or utensil sharing policies.

Training for school staff is needed not only for treating appropriately an allergic reaction but also for preventing and identifying anaphylaxis timely. Furthermore, a whole school approach should be adopted, empowering all staff members to have a role in managing pupils’ allergies and supporting them while at school, as opposed to being directed just at those deemed responsible. There is a delicate balance between supporting and overburdening schools, so further research, employing qualitative techniques, is required to identify what interventions or training methods would be effective in the school setting to tackle this important public health issue. This will inform an action plan, led by schools and supported by health professionals, patients and parents in order to improve school preparedness and restore confidence in the management of severe allergic reactions.

This survey is novel in that it used a questionnaire based on guidelines published by accredited organisations for measuring preparedness in schools. The questionnaire content was further checked for validity and readability by a group of various professionals. However, some limitations must be acknowledged. The response rate was lower than expected yet the characteristics of responders and non-responders were fairly similar. School staff can be overwhelmed by the number of surveys they are asked to participate in. As the survey was fairly detailed and required sourcing of information, this may have discouraged some schools to complete it. The survey was sent in paper format, requiring the respondent to post it back to the researcher, which may have further affected the response rate. A combined approached using online surveys may have improved the response rate.

The survey results reflect the status of schools’ staff preparedness just after the publication of national and international guidelines in 2014, giving an indication of the degree to which the guidance was implemented between 2015 and 2016. Whether the same findings persist needs to be explored. Since the time of this survey, further barriers towards school preparedness have arisen with the reduction in school nurses and limited resources for regular face-to-face training [14, 25, 27]. It is therefore likely that the current preparedness level has decreased, although further research is warranted and currently underway.

We also surveyed a single UK site, and therefore conclusions may not be extrapolated to the other setting across the country. Nonetheless, the results seem to be in agreement with previous surveys from other UK regions [23, 30]. Finally, the survey was not anonymised, and this may have introduced social desirability bias or over-reporting school preparedness. Should this be the case, the true figures of school unpreparedness would be even lower than the ones presented here.

## Conclusion

This survey revealed areas of good practice and areas that need improvement in order to create a safer school environment for children with existing and unknown risk of anaphylaxis. The responsibility for a safe school does not lie only with school staff; this should be shared between all stakeholders with school personnel, allergy specialists, community health professionals, patient associations, allergic pupils and their parents working collaboratively within clear legislation. There is now a need to resurvey school preparedness, to ascertain whether published guidelines on anaphylaxis management for schools have any impact. In view of frequent requests from schools for training and guidance and especially after the recent episodes of fatal anaphylaxis in school, staff training in anaphylaxis needs urgent review [27].

We strongly believe that working together in developing a national policy that addresses the holistic needs of schools and the allergic pupil and offers implementation support could reduce deficiencies and inequalities in school preparedness.

## Abbreviations

AAI: Adrenaline autoinjector
CI: Confidence intervals
LEA: Local education authorities
PAAP: Personalised allergy action plan
